# Evaluation of Carnitine Status in Postnatal Piglets from Sows Fed Clofibrate

**DOI:** 10.3390/cimb47121004

**Published:** 2025-11-29

**Authors:** Brandon Pike, Jinan Zhao, Julie A. Hicks, Tim Boston, Hsiao-Ching Liu, Jack Odle, Xi Lin

**Affiliations:** Laboratory of Developmental Nutrition, Department of Animal Sciences, North Carolina State University, Raleigh, NC 27695, USA; bepike2@ncsu.edu (B.P.); jnzhao@zinpro.com (J.Z.); jahicks3@ncsu.edu (J.A.H.); teboston@ncsu.edu (T.B.); hc_liu@ncsu.edu (H.-C.L.); jodle@ncsu.edu (J.O.)

**Keywords:** carnitine, clofibrate, maternal, piglets, sow

## Abstract

Milk carnitine content decreases quickly with lactation days and is accompanied by PPARα downregulation. This study aimed to investigate the effects of the maternal supplementation of the PPARα agonist, clofibrate, on milk carnitine content and carnitine status in neonates during lactation. Pregnant sows (n = 27) were fed diets containing either 0, 0.25, or 0.5% clofibrate from d107 gestation to d7 of lactation. Carnitines were determined in milk on d1, 3, 5, 7, 10, 14, and 19 post farrowing and in the plasma, intestine, and liver of piglets on d1, 7, 14, and 19. Milk carnitine decreased quadratically with lactation days (*p* < 0.0001). Hepatic and intestinal carnitines increased with postnatal age (*p* < 0.05). Correlations between levels were detected between milk and plasma (R^2^ = 0.5, *p* < 0.0001) and milk and intestine (R^2^ = 0.23; *p* < 0.05). Clofibrate increased BBH expression (*p* < 0.05) and tended to increase OCTN2 expression (*p* = 0.055) in intestine and *TMLH* in liver (*p* = 0.059). Hepatic *ALD* and *TMLH* increased (*p* < 0.0005) with postnatal age. However, gene modification had no effect on plasma and mucosa carnitine concentrations. We conclude that changes in carnitines within plasma and mucosa are mostly driven by milk carnitines during postnatal development.

## 1. Introduction

L-carnitine plays a critical role in the metabolic functions of normal neonatal growth and development, especially in energy production and detoxification via acyl-residue removal [1]. The primary source of carnitine for fetuses and neonates is from the mother via placental transfer and breast milk uptake due to the limited capacity of neonates to synthesize carnitine de novo [2]. Though maternal placental transfer and milk uptake are very important, the functional capacity to synthesize carnitine has also been observed in the human fetal kidney, liver, and spinal cord [3]. Results from human studies have shown that the carnitine reserves in full term newborns are about 25–50% of those in adults [4] and that the reserves in preterm infants are even lower [5]. Low carnitine status was also reported in neonatal animals such as rats [6], cats [7], dogs [8], and pigs [9], suggesting that carnitine reserves may not be sufficient for the optimal metabolic requirements of neonates. Indeed, poor feeding, low blood sugar, lethargy, and muscle weakness in human infants can be associated with low carnitine status at birth [10]. In addition, low carnitine status affects energy metabolism due to its crucial role in transporting fatty acid (FA) into mitochondria. Relative carnitine deficiency impairs FA oxidation, leading to insufficient energy generation for growth and development, particularly for neonates who have limited energy reserves. For example, results from our recent studies with neonatal piglets, a species with only 2% body fat at birth, showed that supplementation of carnitine via milk replacer/feeding to sow facilitates FA oxidation in the liver, kidney, and intestine tissues [11,12,13]. These results suggest that neonatal piglets might not absorb sufficient carnitine via milk to support robust FA oxidation. Sufficient carnitine intake is critically important for domestic piglets to obtain sufficient energy from the metabolism of milk fat, and impairments may contribute to the high mortality rate of newborn piglets.

The uptake of carnitine during the suckling period depends on its concentration in milk, which is dependent upon the capacity of maternal carnitine synthesis and the availability of carnitine transporters. Milk carnitine levels in humans and other mammals vary greatly between species, lactation stage, and diets [14,15]. The effect of dietary carnitine on maternal and mammary gland metabolism has been described [16]. However, the effect of lactation stage on milk carnitine and the carnitine status of suckling neonates has not been well evaluated, in part because of the dynamic changes over time. These changes are closely related to both the ability to synthesis carnitine as well as the transfer (or uptake) efficiency of carnitine. Both carnitine synthetic enzymes and organic cation transporters (OCTNs), which play important roles in carnitine synthesis and transfer, are regulated by peroxisome proliferator-activated receptors alpha (PPARα), a ligand-activated transcription factor of a nuclear receptor [17]. It has been reported that blocking PPARα activity in mice increased long-chain carnitine while decreasing short-chain carnitine in the plasma. PPARα inhibition also caused 40–50% reductions in free carnitine in plasma and tissues, subsequently resulting in defective fat oxidation [18]. In addition, results from fasted rats show increased carnitine concentration in the liver by an upregulation of enzymes of hepatic carnitine synthesis and novel OCTN2, mediated by the activation of PPARα [19]). The administration of clofibrate to rats (0.5%) increased carnitine absorption in the small intestine, likely due to the observed upregulation of OCTN2 mediated by the activation of PPARα [20,21]. Beyond the effects in local tissues, PPARα mediates changes in whole-body carnitine homeostasis in mice [22].

From this, it is inferred that PPARα activation is important for maternal carnitine synthesis and transport to provide sufficient carnitine in milk. However, in rats, PPARα is downregulated during lactation [23]. Decreased expression of the genes involved in hepatic carnitine synthesis and uptake was observed, irrespective of litter size. This downregulation of PPARα may serve to conserve energy and metabolic substrates for milk production in the mammary gland [23]. Even so, it is unknown whether this downregulation affects milk carnitine concentration, which results in insufficient carnitine uptake during suckling and ultimately impacts normal fat metabolism [24]. Liver carnitine is very high during the first 2–3 days postpartum and falls as nursing continues [6]. Results from a suckling guinea pig showed that carnitine concentration in the intestine peaked at d3 and decreased to the near-adult level at d7 after birth [25]. Therefore, we hypothesized that the stimulation of PPARα in pregnant sows might increase milk carnitine concentration and boost intestinal carnitine uptake in suckling piglets via the promotion of gene expression associated with carnitine synthesis and transporters in the liver and intestine. Carnitine concentrations in organs depend upon dietary intake during the early suckling period, in which the small intestine is recognized as a considerable proportion of the carnitine pool [24]. We also hypothesized that carnitine synthesis and transporters in the liver and intestine would be promoted by maternal clofibrate. To test our this, we administrated clofibrate, a PPARα agonist, to the pregnant sows during late gestation and early lactation and evaluated milk carnitine status and its influence on carnitine levels of the intestine, plasma, and liver in piglets. 

## 2. Materials and Methods

### 2.1. Animals and Treatment 

Animal care and all experimental procedures were approved by the Institutional Animal Care and Use Committee (IACUC) at North Carolina State University, IACUC ID 16-142. The full design of the animal study was described previously [11]. Briefly, 27 gestating sows in 3 blocks with 9 sows in each block (blocked by farrowing time) were divided into three groups and fed standard commercial diets, as described by Zhao and Kim [26], supplemented with three levels of clofibrate: 0% (control), 0.25% (clof 0.25), and 0.5% (clof 0.5) of feed based on previous studies [11,27]. Clofibrate was supplemented to the treated sows pre-dissolved in 15 mL of ethanol and the control sows received 15 mL of plain ethanol only. The sows were fed twice daily following the standard for the gestation and lactation of sows and the ethanol, with or without clofibrate, was mixed well with a small amount of feed and was given to the sows before feeding each morning. The supplementation started from one-week pre-farrowing and extended one-week post-farrowing. Cross fostering of the piglets was arranged within treatment groups after farrowing to balance the litter size to 10-12 pigs. Following oxytocin (1cc in the vulva tissue) injection, sow milk samples were collected manually from the proximal 4 teats on d1, 3, 5, 7, 10, 14, and 19 during the lactation period and were stored at −80 °C until analysis. Sow reproductive performance and piglet growth performance were recorded and evaluated [11].

One piglet with an average body weight from each litter was sampled on d1, 7, 14, and 19. They were euthanized via American Veterinary Medical Association-approved exsanguination while under deep (5%) isoflurane anesthetization. Blood samples were collected via heart puncture immediately prior to euthanasia, and plasma were obtained by centrifuging the blood samples at 2000× *g* for 20 min at 4 °C and stored at −80 °C until analysis. Liver samples from left lobe and intestinal mucosa samples from both the proximal and distal ends of the small intestines in 30-cm segments were collected and frozen in liquid N_2_ immediately and stored at −80 °C until analysis. 

### 2.2. Samples Analysis

#### 2.2.1. Carnitine Analysis

Free carnitine and acyl-carnitine in the milk, plasma, tissues, and feed were analyzed as described by McGarry and Foster [28] with slight modifications, as described by Bhuiyan et al. [29]. The analyses were performed with 100 mg of liver and intestinal mucosa, 100 µL of plasma and milk, and 100 mg of feed. Briefly, the samples were homogenized in ice cold HCLO_4_ (1M) using a polytron electric homogenizer (VWR VDI25, Radnor, PA 19087, USA). The homogenate was then centrifuged at 10,000× *g* for 5 min and the supernatant was collected. The pellet was then washed with 0.1M HClO_4_ and recentrifuged at the same speed. The two supernatants were combined and neutralized with 1M KOH for free carnitine analysis. Acyl-carnitines were analyzed after alkaline hydrolysis with KOH at 60 °C for 60 min. Analysis was conducted in HEPES–EDTA buffer (pH = 7.3) with 25.5 nmol [1-^14^C]acetyl-CoA (37 kBq/μmol), 2 μmol N-ethymaleimide, and 1 IU carnitine acetyltransferase at 25 °C for 30 min. Radioactivity was determined by liquid scintillation after the labeled acetyl-carnitine from the analysis was separated on a column packed with resin (AG 1×8, 100–200, chloride form; Bio-Rad, Richmond, CA 92195, USA). All samples were prepared and analyzed in duplicate.

#### 2.2.2. Gamma-Butyrobetaine Hydroxylase Assay

The protein of γ-butyrobetaine hydroxylase (BBH) was determined in the homogenate from the frozen mucosa samples using the ELISA Kit MBS2903999 (MybioSource, Inc., San Diego, CA 92195, USA) following the manufacturer’s instructions.

#### 2.2.3. RNA Isolation and RT-qPCR

Total RNA was isolated from the mucosa and liver samples using Tri reagent, TRIZOL (13) with 50 mg of the frozen samples. The quantification, quality control, and the cDNA synthesis were performed with a Nanodrop spectrophotometer (ND-1000 ThermoFisher, Wilmington, DE 19810, USA), and 0.8% agarose gel was run post DNase treatment; the Super Script III Reverse Transcriptase was used as described previously (13). Primers for RT-qPCR (Table S1) were created using BLAST Primer Designer (https://www.ncbi.nlm.nih.gov/tools/primer-blast/, accessed on 27 October 2025). The RT-qPCR reaction and the CT values were normalized to a housekeeping gene (RPL9), and a plate normalizer was included on each plate to account for run differences. The relative changes in gene expression (normalized to newborn pigs) were calculated from the real-time PCR data using the 2−^ΔΔC^_T_ method, where ΔΔC_T_ = (C_T_. _Target_ – C_T_.RPL9)_age X_ – (C_T_. _Target_ – C_T_._RPL9_)_age 0_ [30]. 

### 2.3. Chemicals

All chemicals were sourced from Sigma-Aldrich, Inc (St. Louis, MO 63103, USA). Clofibrate was sourced from Cayman Chemical (Ann Arbor, MI 48108, USA), Superscript was from Thermo Fisher Scientific (Waltham, MA 02451, USA), TurboDNase was from Ambion, and SYBR green was from BioRad (Hercules, CA 94547, USA).

### 2.4. Statistical Analysis

Data from biological and gene expression analyses of piglets were subjected to a 3 (control, 0.25, and 0.5% clofibrate) × 7 (d1, 3, 5, 7, 10, 14, and 19) for milk and × 4 (d1, 7, 14, and 19) factorial randomized complete block design for tissues using the General Linear Model (GLM) procedure of SAS (SAS software 9.4; Cary, NC 27513, USA). The least square means (Lsmeans) were calculated and the interactions between clofibrate x age were tested for piglets. The effect of maternal clofibrate was also tested using contrast of control vs. clofibrate. Polynomial contrasts were conducted for carnitines to detect the linear and quadratic effects of lactation days and postnatal ages. The difference and correlation between plasma and tissues were evaluated using the SAS procedure (PROC CORR) for correlation. The data from main effects were reported only if the interaction was not significant. Data are presented as Lsmeans ± standard error mean (SEM) if not specified otherwise. Differences were considered significant when *p* < 0.05 and trends were noted when 0.05 < *p* < 0.1.

## 3. Results

### 3.1. Carnitine Concentration in Sow Feed

Free carnitine, acyl-carnitine, and total carnitine concentrations in sow feed samples from three representative samples averaged 0.034 ± 0.002, 0.079 ± 0.0040, and 0.113 µmol/g feed (5.3 mg, 12.7 mg, and 18.2 mg/kg feed).

### 3.2. Carnitine Concentration in Sow Milk

No significant impact of maternal clofibrate on carnitines (Table 1) was detected (*p* > 0.1). However, the average acyl-carnitine concentration (0.46 nmol/mg) in clof 0.25 and clof 0.5 was 12% higher than that (0.41 nmol/mg) in clof 0 and total carnitine concentration (1.27 nmol/mg) was 11% higher than that (1.14 nmol/mg) in clof 0 (*p* = 0.089). Clofibrate also had no impact on the distribution between carnitine and acyl-carnitine and the ratio of acyl-carnitine/free carnitine (Ac/Fc) tended to increase (*p* = 0.094). 

Lactation days (Table 1) had impacts on milk free carnitine, acyl-carnitine, and total carnitine (*p* < 0.0001). Significant linear and quadratic responses of carnitines to lactation days were detected (*p* < 0.0005). Both concentrations of free and acyl-carnitine were high at birth and decreased as lactation progressed. The concentrations reached the lowest level at 10 days and then gradually increased. The total carnitine followed the same pattern as free and acyl-carnitine. However, the percentage of free carnitine increased, and the percentage of acyl-carnitine decreased quadratically (*p* < 0.01). The Ac/Fc followed the same pattern as a percentage of acyl-carnitine but tended to be higher on d5 and d14 than d10 (*p* = 0.075). 

### 3.3. Carnitine Concentration in Plasma of Piglets

Maternal clofibrate (Table 2) had no impact on free, acyl-, and total carnitine concentration in the plasma of piglets (*p* > 0.1), but the percentage of free carnitine tended to decrease, and the percentage of acyl-carnitine increased by maternal supplementation of clofibrate (*p* = 0.08). Postnatal age had a great impact on the concentration of free (*p* < 0.05), acyl- (*p* < 0.0001), and total carnitine (*p* < 0.01). The plasma’s free, acyl-, and total carnitine measured during the first 7 days were on average 1.4-, 2.3-, and 1.5-fold of that measured after 7 days. Moreover, the acyl-carnitine concentration was 63% higher on d1 than d7. The percentage of free carnitine increased, and the percentage of acyl-carnitine decreased after d1 (*p* < 0.001). The Ac/Fc followed the same pattern as the percentage of acyl-carnitine (*p* < 0.001). A linear response was detected for free and total carnitine. Both linear and quadratic responses were detected for acyl-carnitine and the percentage of free carnitine, acyl-carnitine, and Ac/Fc (*p* < 0.05). 

### 3.4. Carnitine Concentration in Liver of Piglets

Maternal clofibrate (Table 3) had no impact on the free, acyl-, and total carnitine concentration in the liver of piglets (*p* > 0.1), but the average free carnitine from clof 0.25 and clof 0.5 tended to be higher than that from clof 0 (*p* = 0.08). No maternal clofibrate influences were detected for the percentage of carnitines and the Ac/Fc (*p* > 0.1). The concentrations of free (*p* < 0.005), acyl- (*p* < 0.1), and total carnitine (*p* < 0.05) were impacted by the postnatal age (Table 3). The free carnitine values measured on d7 and d14 were on average 36% higher than that measured on d1 but were not different than that from d19. The acyl-carnitine concentration measured from d7 was 32% higher than that from d19, but no difference was detected at d1 and d14. Total carnitine was 24% higher on d7 than d1 and d19 but no difference was observed between d7 and d14. A quadratic response to postnatal age was detected for free, acyl-, and total carnitine (*p* < 0.05). The percentage of free carnitine increased, and acyl-carnitine decreased linearly (*p* < 0.05), while the Ac/Fc followed the same pattern as the acyl-carnitine. 

### 3.5. Carnitine Concentration in Intestinal Mucosa of Piglets

Maternal clofibrate had no impact on free, acyl-, or total carnitine concentration, the percentage of free or acyl-carnitine, nor the Ac/Fc in the intestinal mucosa of piglets (*p* > 0.1). However, the concentrations were significantly impacted by the postnatal age (Table 4). The free carnitine concentration increased with age after d7, and the concentrations measured on d14 and 19 were on average 40 and 95% higher than those measured on d1 and d7 (*p* < 0.0001). The acyl-carnitine measured on d14 and d19 was on average 65% higher than that measured on d7 (*p* < 0.001) but was not different from d1. The total carnitine concentrations measured from d14 and 19 were on average 56% higher than that from d1 and d7 (*p* < 0.0005). The percentage of free carnitine was higher from d19 than d1 and the percentages of acyl-carnitine and the Ac/Fc were higher on d1 compared to all other days (*p* < 0.05). Linear responses to the postnatal age were detected for free carnitine, acyl-carnitine, and total carnitine concentrations as well as for the percentage of free and acyl-carnitine (*p* < 0.05).

### 3.6. Coefficient of Correlations Between Maternal Milk Carnitines and Carnitines in Tissues of Piglets

Significant positive correlations (Table 5) were detected between milk and plasma for free (R^2^ = 0.49; *p* < 0.0001), acyl- (R^2^ = 0.37; *p* < 0.0005), and total carnitine (R^2^ = 0.50; *p* < 0.0001). The positive correlation between milk and intestinal mucosa was also significant for acyl- (R^2^ = 0.22; *p* < 0.05) and total carnitine (R^2^ = 0.23; *p* < 0.05) and tended to be significant for free carnitine (R^2^ = 0.20; *p* = 0.06). No significant correlations between milk and liver were observed for free carnitine (*p* > 0.1). Acyl-carnitine and total carnitine in the milk tended to be positively correlated to hepatic acyl-carnitine (R^2^ = 0.203; *p* = 0.058) and total carnitine (R^2^ = 0.195; *p* = 0.069). No correlations were detected between plasma carnitines and mucosa and liver or between mucosa and liver (*p* > 0.1). In addition (Table S2), no correlations were observed for Ac/Fc between milk and plasma and between milk and mucosa (*p* > 0.1), but a significant correlation was detected between milk and liver (R^2^ = 0.26; *p* < 0.05). Correlations for Ac/Fc were also observed between plasma and intestine (R^2^ = 0.32; *p* < 0.005) and between plasma and liver (R^2^ = 0.33; *p* < 0.005). No correlation was observed between the intestine and liver (*p* > 0.01). 

### 3.7. BBH Protein Concentration in Intestinal Mucosa

**Figure 1:**
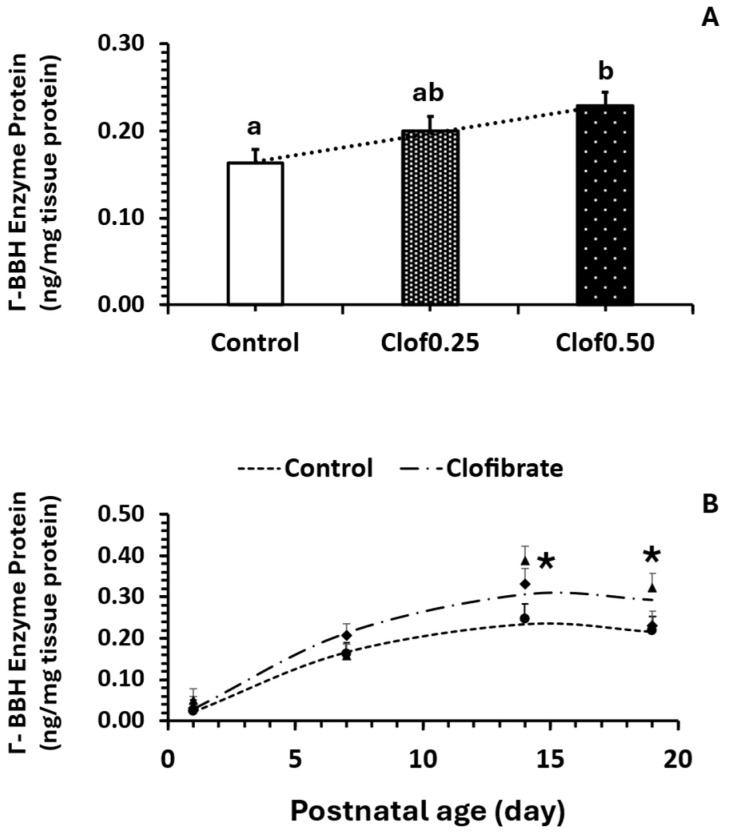
Effect of maternal clofibrate (**A**) and postnatal age (**B**) on intestinal BBH protein. Clof: clofibrate and 0, 0.25, and 0.5 represent the diet level of clofibrate (% in the feed). Values are least square means ± SEM. ^ab^ The least square means lacking a common superscript differ (*p* < 0.05) in (**A**). * Indicates the difference between control and clofibrate (*p* < 0.05) in (**B**).

BBH protein was impacted by the clofibrate dosage (Figure 1A; *p* < 0.05) and enzyme protein (ng/mg total tissue protein) was 25 and 44% higher from clofibrate 0.25% (0.2) and clofibrate 0.5% (0.23) than the control (0.16). Postnatal age (Figure 1B) also significantly impacted BBH protein (*p* < 0.0001) concentration. Protein concentration increased with age following a quadratic response in which the highest response was observed on d14 in the suckling piglets from both clofibrate-treated and untreated sows. 

### 3.8. Gene Expression (RT-PCR) in Intestinal Mucosa and Liver

Maternal supplementation of clofibrate (Table 6) had no impact on the expression of selected FA oxidation-associated genes in the intestinal mucosa of pigs (Table 6; *p* > 0.05) except for tending to increase *OCTN2* expression (*p* = 0.052). Postnatal age also had no impact on all measured genes (*p* > 0.1). Interaction between maternal clofibrate and postnatal age tended to be significant for *BBH* (*p* = 0.089). *BBH* enrichment was greater from piglets born to sows fed 5% clofibrate than controls on d1 and was higher on d7 than on d1.

The expression of genes *ALD* and *BBH* (Table 6) in the liver was not affected by maternal supplementation of clofibrate (*p* > 0.1) but the expression of *TMLHE* tended to increase (*p* = 0.076). The increase was affected by maternal clofibrate levels (*p* = 0.059). The enrichment was 41% and 45% higher in piglets from 0.5% clofibrate than control or 0.25%, respectively. In addition, the expression of *ALD* and *TMLHE* increased in piglets with postnatal age (*p* < 0.001). *ALD* increased by 77% at *d*19 and *TMLHE* increased by 3.9-fold at d7 and 14 compared to d1. *BBH* expression also tended to increase with postnatal age (*p* = 0.066). A thread of interaction between clofibrate and age observed in intestinal mucosa for BBH was observed (*p* = 0.089).

## 4. Discussion

### 4.1. The Effects of Maternal Supplementation of Clofibrate on Carnitine Concentrations in Sow Milk Throughout Lactation

**Figure A1:**
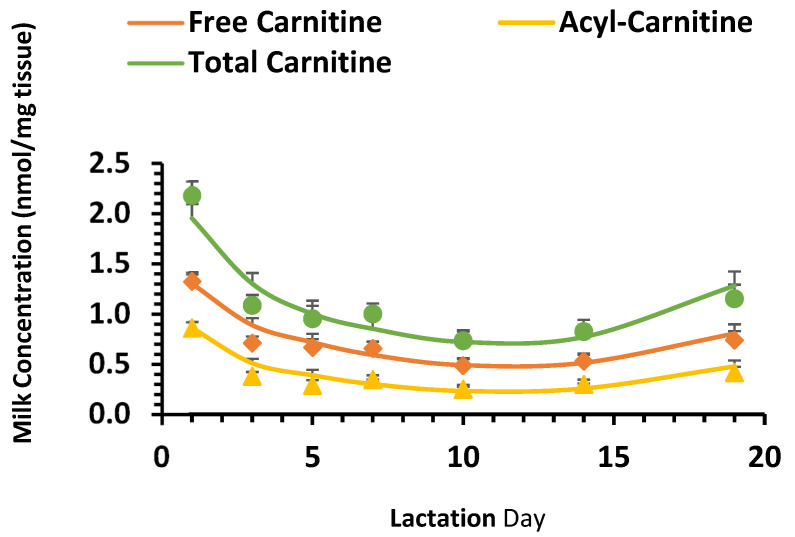
Dynamic changes in carnitines during the lactation period. Values are least square means ± SEM.

There have been several previous studies evaluating milk carnitine concentration in sows; however, unlike the present study, they focused only on a single day of lactation [31]). The total concentration of carnitine measured in our study was higher than that in milk measured on d11 by Ramanau et al. [31]. Considering that the data in sow milk reported previously was from a single day mid-lactation and from sows of different breeds, the observed differences could be due to variations in dietary carnitine level, the capacity of carnitine synthesis, and metabolic (physiological) status. The reproductive productivity of sows such as litter size and milk production could also affect milk carnitine concentrations. To our knowledge, this is the first study to examine milk carnitine repeatedly over the course of the entire lactation period. The data presented here greatly improves our knowledge of the dynamic changes during the lactation period (Figure A1). The strong correlations between sow’s milk, piglet’s plasma, and intestinal mucosa, as well as the total measured (plasma+ intestinal mucosa + liver) carnitines suggest that the carnitine in sow’s milk plays an important role in carnitine intake and metabolic development in neonatal piglets.

Maternal treatment with clofibrate during the last week of gestation and the first week of lactation had no impact on milk free carnitine level but tended to increase (*p* = 0.089) milk acyl-carnitine and total carnitine concentrations. Very limited information about the effect of clofibrate dosage and treatment duration on milk carnitine concentrations can be found in the literature. The subdued response that we observed could be due, at least partially, to the duration of clofibrate treatment and/or the efficiency of clofibrate absorption, which is supported by the result from milk clofibrate analysis. 

No clofibrate or its metabolites were detected in the milk samples collected in our study. Clofibrate transfer from mother to offspring via milk was observed in rats [32]; however, clofibrate (500 mg kg^−1^) was administered to the lactating rats via intraperitoneal injection rather than provided as a feed additive as in the present study. In addition, reports from human studies indicate that infant drug exposure via breast milk is low and the relative infant dose expressed as a percentage of the similarly adjusted mother’s dose is <10% for most drugs [33]. This suggests that the dietary clofibrate level used in our study may have been lower than was needed to produce a substantial response in milk clofibrate concentration. 

The carnitine concentrations in milk were significantly influenced by the stage of lactation. Free carnitine, acyl-carnitine, and total carnitine follow the same nonlinear pattern (Figure A1), which is the highest at d1, then decreases after d1, with the lowest levels at d10. Similar patterns were reported in human milk; the concentration of carnitine decreased by about 50% by postpartum d40–50 [14,34]. The changes through lactation were not associated with diet as this remained constant during the lactation period, although feed intake per day could vary. The carnitine levels were examined previously in sow colostrum on d1 and milk on d7 [35] and the total carnitine concentration in colostrum was 1.54-fold of that in milk on d7. Consistent with Birkenfeld’s report [35], the total carnitine measured on d1 was 1.5-fold of that on d7 in our study. With a piglet’s limited capacity for the de novo biosynthesis of carnitine [36], it would be expected that the levels of milk carnitine are higher at birth and decrease with lactation days due to the downregulation of PPARα in sows [23]. 

A broad range of acyl-carnitine concentrations (13–47%) have been reported in milk from different species [15]. It is generally believed that variation is related to maternal systemic and/or breast metabolic status. The mild increase in acyl-carnitine and Ac/Fc in the milk of clofibrate-treated sows appeared to be associated with maternal/mammary metabolic status, implying that a metabolic change might occur in mammary tissue after clofibrate supplementation. However, there was no detectable interaction between clofibrate and lactation days. Consistent with the acyl-carnitine concentration, the percentage of acyl-carnitine decreased while the percentage of free carnitine increased quadratically as lactation progressed. Ac/Fc followed the same pattern. These results are in agreement with the results observed in other mammals; the maternal metabolic rate is usually the highest immediately postpartum and gradually decreases as lactation progresses through mid and late stages [37]. 

### 4.2. The Effects of Maternal Supplementation of Clofibrate and Lactation Days on Carnitine Concentrations in Plasma, Liver and Intestinal Mucosa of the Suckling Piglets

**Figure A2:**
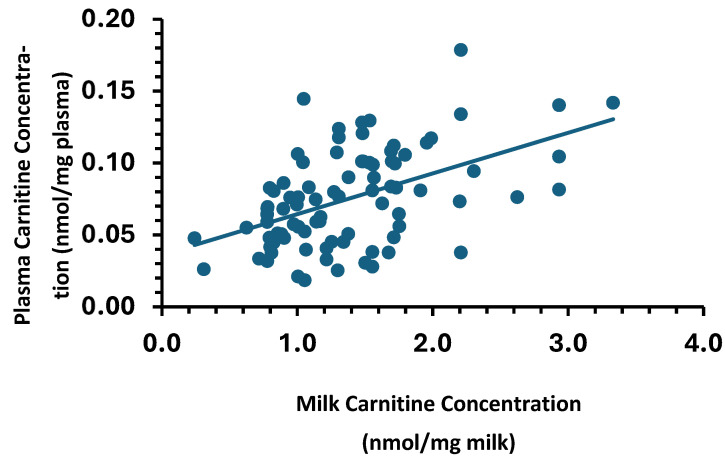
Correlations between maternal milk carnitines and carnitines in tissues of piglets.

The plasma levels of carnitine and acyl-carnitine were similar as that reported by Lyvers Peffer et al. [38] but were higher than that reported by Birkenfeld et al. [35] and Kaup et al. [39]. Compared to other species, the plasma carnitine concentration is similar to that of the breast-fed human infant [40], higher than that of rabbits [41] and rats [42], and lower than piglets receiving milk formula composed primarily of whey [13]. This difference illustrates both inter-species differences as well as the differences in concentrations between milk sources. No differences were detected in the plasma carnitine levels or its distribution and Ac/Fc in piglets from sows with or without the supplementation of clofibrate. However, the results showed that the percentage of free carnitine tended to decrease, with the percentage of acyl-carnitine increasing by maternal clofibrate, suggesting that clofibrate could affect the plasma carnitine component by altering the metabolic status. Additionally, the carnitine in all forms examined decreased with postnatal age. Linear and/or quadratic responses to postnatal age were detected in carnitine, acyl-carnitine, and total carnitine. These observations are consistent with the results reported previously [43]. The changes followed the same pattern as observed in sow milk. The correlation assay indicated that the concentrations of carnitine, acyl-carnitine, and total carnitine are closely associated with the levels in sow milk (Figure A2), demonstrating that plasma carnitine status in neonates was affected by the status of the mother’s milk. However, the concentration of carnitine in maternal milk was much higher than that in the plasma of the piglets. Especially Ac/Fc, which was five-fold higher in sow milk than in piglets’ plasma, suggesting that absorption might differ for carnitine and carnitine esters, possibly due to differing affinities of carnitine transporters. The plasma percentage of free carnitine in total carnitines and Ac/Fc are considered to be markers of carnitine deficiency and insufficiency [44], respectively. It was suggested that the percentage of free carnitine being less than 70% indicated carnitine deficiency and Ac/Fc being greater than 0.40 indicated carnitine insufficiency. Although there were differences in the percentage of free carnitine and Ac/Fc at different postnatal stages, all measured values for the percentage of free carnitine were higher than 70% and for Ac/Fc were lower than 0.40. These results imply that carnitine status and FA oxidation were typical of postnatal development. 

Numerous studies have examined the intestinal ability to absorb carnitine and its relationship to the expression of *OCTNs* within the intestines, but very few studies have examined the carnitine levels present in the intestine mucosa and their changes throughout postnatal development. A previous study with rats showed that carnitine concentration in the mucosa of the proximal small intestine was 0.31 (nmol/mg wet tissue) in a fasting state and 2.5 nmol after enteral loading with 5 mL of a 2 mM carnitine solution [42]. A study with suckling guinea pigs showed that carnitine concentration peaked after 3 days (0.79) and ranged from 0.35 to 0.91 nmol/mg from d1 to d29 [25]. Carnitine content of the small intestinal mucosa of rats decreases postnatally, reaching adult levels at the time of weaning [45]. In agreement with previous findings in other mammals, the total concentration of carnitine measured in the intestinal mucosa of piglets on average was 0.86 (nmol/mg wet tissue), from our previous work [13] and in this study. Maternal supplementation of clofibrate had no impact on carnitine content, and similar results were also observed in our previous work with one-week-old piglets receiving milk formula with and without supplementation of clofibrate [13]. Although the influences of maternal clofibrate on intestinal carnitine concentration were not detected, we noticed that the expression of *OCTN2*, the primary carnitine transporter, tended to increase in pigs from sows treated with clofibrate (*p* = 0.052) and was on average 93% higher than that from control sows, consistent with the result observed in rats [20,21]. In addition, the key enzyme protein of carnitine synthesis, BBH, increased linearly with maternal clofibrate dose. These results suggest that PPARα, indeed, played a regulatory role in intestinal uptake and synthesis of carnitine. Similarly to rodents [46], piglets might be capable of synthesizing carnitine in the intestinal mucosa during the suckling period. In support of this, we examined the mucosa expression of genes associated with carnitine synthesis such as *ALDH9A1*, *BBH*, and *TMLH* during the suckling period. We found that *ALDH9A1* and *TMLH* had no change and maintained a low level compared to newborns, while the expression of *BBH* remained at a high level throughout the lactation period and was influenced by maternal clofibrate (*p* = 0.09). These findings imply that carnitine may not be synthesized from lysine, but we speculate it may be obtained by hydrolyzing 4-trimethlammoniobutanoic acid in the intestinal mucosa. Nonetheless, it is most likely that endogenously synthesized carnitine is very limited in the intestinal mucosa during the suckling period compared to the liver and kidney [47], stressing the importance of milk as the primary source of carnitine. 

Carnitine concentration increased significantly after one week, although the impact of clofibrate on concentration was limited. The increase is likely not related to an increase in transporters as we did not detect significant changes in *OCTN2* gene expression with respect to age. It has been shown in 4-week-old piglets that the level of carnitine absorption at the proximal end of the intestines can reach as high as 95% [48], and even with high levels of carnitine supplementation, the absorption rate was still around 90%, suggesting that OCTN2 might not be a limiting factor for carnitine absorption. Moreover, a correlation between milk and intestine but not between intestine and plasma carnitine concentration was detected, emphasizing the role of the small intestine in carnitine absorption. However, BBH protein increased with the postnatal age. This link between the carnitine levels in the intestine might need to be investigated further as 4-trimethylammoniobutanoic acid can be produced by intestinal microbes [49]. 

The total carnitine concentration, in general, was similar to previous work from our laboratory [38], and others [19,50] for neonatal pigs. Concentrations were also similar to reports for rats [51] and dogs [8]. However, the acyl-carnitine concentrations varied over a wide range among these studies, demonstrating that the carnitine status in liver is sensitive to alterations to hepatic metabolism. The proportion of acyl-carnitine measured was over 50% and was higher on d1 than other days. This is consistent with increased FA oxidation, in which acetyl-carnitine is the main component of the acid-soluble products [11]. The liver is the predominant site of carnitine biosynthesis in the sow with the kidney providing a relatively small amount, while the contribution of other tissues is negligible [50]. Evidence from early studies showed that carnitine synthesis in the liver and kidney and uptake in the hepatocyte could be increased by the activation of PPARα [19,52]. Indeed, long-term clofibrate administration markedly increased the concentration of carnitine as well as the activity of mitochondrial carnitine palmitoyl-transferase in the liver [53]. Consistent with these early findings, we also observed that liver carnitines increased greatly in newborn pigs when fed with clofibrate directly [13]. However, here, only a small increase in free carnitine induced by maternal clofibrate supplementation was observed. Regarding the status of carnitines in milk, we postulate that the lack of response was related to the maternal clofibrate dosage, the lapsed time between supplementation and sample collection, and low efficiency of clofibrate transfer via milk. This was consistent with the tendency for an increase to *TMLH* at the higher dose of maternal clofibrate. 

The pattern of carnitine changes during the postnatal period looks similar to that in piglets reported by Li et al. [54]. The concentrations increased with postnatal age from d1 to d7, and no further increase was observed after d7, though the increase in acyl-carnitine differed from the level of free carnitine. Significant quadratic responses were detected. The increase in hepatic carnitines seems not to be associated with milk concentrations but is possibly due to increased carnitine synthetic capacity and ingestion, because the milk concentration decreased at the same stage while the measured genes associated with carnitine synthesis such as *TMLH*, *ALD*, and *BBH* as well as the transporter OCTN2 increased [11]. This suggests that endogenously synthesized carnitine could play an important role in the liver where carnitine synthesis and metabolism exhibit robust activity with an increase in capacity as they age [55,56]. This likely accounts for the trends seen in the liver of these piglets. As the sow’s supply of carnitine reduced in the milk, the biosynthesis capacity of the piglets increased to compensate for the difference. The sow was still supplying ample carnitine and the piglet’s own capacity to synthesize carnitine was beginning to develop. 

## 5. Conclusions

Milk carnitine decreases quadratically with lactation days. Maternal supplementation of clofibrate tended to increase acyl-carnitine in milk and influence the percentage of acyl-carnitine in the plasma and intestinal mucosa of piglets. There is a correlation between plasma and milk and a correlation between intestinal mucosa carnitine and milk in carnitine concentrations but not between liver and milk carnitines, suggesting that the intestine plays an important role in carnitine transport from milk to plasma. 

## Figures and Tables

**Table 1:** Effect of maternal supplementation of clofibrate during gestation and lactation on milk carnitines *.

	PPARα Activator (Clof) *						Lactation (Day)								Clof * Day
Carnitines	Clof 0	Clof 0.25	Clof 0.5	SEM	*p*-Value	1	3	5	7	10	14	19	SEM	*p*-Value	*p*-Value
	**nmol/mg**						**nmol/mg**								
Free Carnitine	0.736	0.824	0.797	0.041	0.2904	1.179 ^c^	0.854 ^b^	0.831 ^b^	0.773 ^b^	0.554 ^a^	0.592 ^ab^	0.716 ^ab^	0.064	0.0001^+^	0.1573
Acyl-Carnitine ^#^	0.407	0.448	0.471	0.026	0.2038	0.759 ^c^	0.436 ^b^	0.468 ^b^	0.397 ^b^	0.269 ^a^	0.360 ^ab^	0.405 ^b^	0.050	0.0001^+^^	0.1702
Total Carnitine ^#^	1.142	1.272	1.269	0.061	0.2320	1.938 ^d^	1.290 ^c^	1.299 ^c^	1.170 ^bc^	0.823 ^a^	0.952 ^ab^	1.121 ^bc^	0.119	0.0001^+^^	0.1138
		*%*					*%*								
Free- Carnitine	65.06	64.75	62.49	1.036	0.1815	60.66 ^ab^	66.69 ^c^	64.59 ^bc^	66.04 ^c^	67.47 ^c^	59.84 ^a^	63.42 ^ab^	1.578	0.0058^^^	0.2688
Acyl- Carnitine	34.94	35.25	37.51	1.036	0.1815	39.34 ^b^	33.31 ^a^	35.41 ^ab^	33.96 ^a^	32.53 ^a^	40.16 ^b^	36.58 ^ab^	1.578	0.0058^^^	0.2688
Ac/Fc	0.552	0.560	0.682	0.045	0.0944	0.648	0.499	0.548	0.514	0.482	0.671	0.577	0.069	0.0754^^^	0.1883

* Clofibrate was supplemented to sows during the last week of gestation and the first week of lactation. Clof: clofibrate and 0, 0.25, and 0.5 represent the diet level of clofibrate (% in the feed). Ac/Fc: ratio of acyl-carnitine/free carnitine. Values are least square means from each group (*n* = 9) in nmol/mg corrected by the average specific gravity of 1.031. ^abcd^ Means lacking a common superscript differ (*p* < 0.05). *^+^* Denotes linear response and ^ denotes quadratic response (*p* < 0.01). ^#^ Control vs. clof for acyl-carnitine (*p* = 0.0890) and for total carnitine (*p* = 0.0888).

**Table 2:** Effect of maternal supplementation of clofibrate during gestation and lactation on plasma carnitine in suckling piglets *.

	PPARα Activator *						Age (Day)					Clof * Age
Carnitines	Clof 0	Clof 0.25	Clof 0.5	SEM	*p*-Value	1	7	14	19	SEM	*p*-Value	*p*-Value
		**nmol/mg**					**nmol/mg**					
Free Carnitine	0.058	0.056	0.062	0.005	0.7321	0.069 ^b^	0.068 ^b^	0.048 ^a^	0.050 ^a^	0.006	0.0346 ^+^	0.9163
Acyl-Carnitine	0.007	0.007	0.008	0.001	0.6626	0.013 ^c^	0.008 ^b^	0.003 ^a^	0.006 ^ab^	0.011	0.0001 ^+^^	0.7411
Total Carnitine	0.065	0.063	0.070	0.006	0.6999	0.082 ^b^	0.075 ^b^	0.051 ^a^	0.056 ^a^	0.022	0.0089 ^+^	0.9011
		*%*					*%*					
Free Carnitine ^#^	90.25	88.15	88.37	0.960	0.2278	85.38 ^a^	90.02 ^b^	91.51 ^b^	88.79 ^b^	1.133	0.0008 ^+^^	0.3088
Acyl-Carnitine ^#^	9.75	11.85	11.62	0.960	0.2278	14.6 ^b^	9.98 ^a^	8.49 ^a^	11.21 ^a^	1.133	0.0008 ^+^^	0.3088
Ac/Fc	0.11	0.14	0.14	0.014	0.3334	0.18 ^b^	0.11 ^a^	0.09 ^a^	0.13 ^a^	0.016	0.0006 ^+^^	0.3944

* Clofibrate was supplemented to sows during the last week of gestation and the first week of lactation. Plasma concentrations were adjusted to nmol/mg using specific gravity (1.022–1.026). Clof: clofibrate and 0, 0.25, and 0.5 represent the diet level of clofibrate (% in the feed). Ac/Fc: ratio of acyl-carnitine/free carnitine. Values are least square means (*n* = 9). The final column is the interaction between clofibrate and age. ^abc^ Means lacking a common superscript differ, (*p* < 0.05), ^+^ denotes linear response, and ^^^ denotes a quadratic response (*p* < 0.01). ^#^ Control vs. Clof (*p* = 0.08) for free carnitine and acyl-carnitine.

**Table 3:** Effect of maternal supplementation of clofibrate during gestation and lactation on liver carnitine status in suckling piglets *.

	PPARα Activator *						Age (Day)					Clof * Age
Carnitines	Clof 0	Clof 0.25	Clof 0.5	SEM	*p*-Value	1	7	14	19	SEM	*p*-Value	*p*-Value
		**nmol/mg Tissue**					**nmol/mg Tissue**					
Free Carnitine ^#^	0.254	0.286	0.296	0.017	0.1779	0.225 ^a^	0.308 ^b^	0.307 ^b^	0.275 ^ab^	0.019	0.0039 ^^^	0.7087
Acyl-Carnitine	0.250	0.267	0.258	0.017	0.7706	0.252 ^ab^	0.294 ^b^	0.265 ^ab^	0.222 ^a^	0.019	0.0709 ^^^	0.9735
Total Carnitine	0.504	0.553	0.554	0.031	0.4226	0.477 ^a^	0.602 ^b^	0.572 ^ab^	0.497 ^a^	0.033	0.0259 ^^^	0.9502
		*%*					*%*					
Free Carnitine	47.5	49.5	50.5	1.482	0.3257	42.89 ^a^	49.29 ^b^	51.00 ^b^	53.45 ^b^	1.721	0.0003 ^+^	0.2002
Acyl-Carnitine	52.5	50.5	49.5	1.482	0.3257	57.11 ^b^	50.71 ^a^	49.00 ^a^	46.55 ^a^	1.721	0.0003 ^+^	0.2002
Ac/Fc	1.26	1.08	1.08	0.100	0.3110	1.61 ^b^	1.08 ^a^	1.01 ^a^	0.86 ^a^	0.117	0.0001 ^+^	0.1043

* Clofibrate was supplemented to sows during the last week of gestation and the first week of lactation. Clof: clofibrate and 0, 0.25, and 0.5 represent the diet level of clofibrate (% in the feed). Ac/Fc: ratio of acyl-carnitine/free carnitine. Values are least square means (*n* = 9) in nmol/mg of fresh tissue. The final column is the interaction between clofibrate and age. ^ab^ Means lacking a common superscript differ, (*p* < 0.05). ^+^ Denotes significant linear response and ^^^ denotes quadratic response. ^#^ Control vs. clof (*p* = 0.08).

**Table 4:** Effect of maternal supplementation of clofibrate during gestation and lactation on intestinal mucosa carnitine in suckling piglets *.

	PPARα Activator *					Age (Day)						Clof * Age
	Clof 0	Clof 0.25	Clof 0.5	SEM	*p*-Value	1	7	14	19	SEM	*p*-Value	*p*-Value
		**nmol/mg Tissue**					**nmol/mg Tissue**					
Free Carnitine	0.105	0.097	0.097	0.009	0.7232	0.076 ^a^	0.074 ^a^	0.105 ^b^	0.144 ^c^	0.006	0.0001 ^+^	0.5555
Acyl-Carnitine	0.181	0.165	0.161	0.014	0.5786	0.144 ^ab^	0.124 ^a^	0.191 ^b^	0.217 ^b^	0.017	0.0012 ^+^	0.8922
Total Carnitine	0.286	0.262	0.258	0.022	0.6130	0.221 ^a^	0.198 ^a^	0.296 ^b^	0.360 ^b^	0.026	0.0002 ^+^	0.7848
		%					%					
Free Carnitine	36.60	36.12	37.80	1.489	0.6988	33.51 ^a^	37.26 ^ab^	36.13 ^ab^	40.46 ^b^	1.730	0.0432 ^+^	0.6292
Acyl-Carnitine	63.40	63.88	62.20	1.489	0.6988	66.49 ^b^	62.74 ^a^	63.87 ^a^	59.54 ^a^	1.730	0.0432 ^+^	0.6292
Acyl/Free	1.78	1.96	1.73	0.144	0.4866	2.23 ^b^	1.79 ^a^	1.82 ^ab^	1.46 ^a^	0.18	0.0144	0.4892

* Clofibrate was supplemented to sows during the last week of gestation and the first week of lactation. Clof: clofibrate and 0, 0.25, and 0.5 represent the diet level of clofibrate (% in the feed). Ac/Fc: ratio of acyl-carnitine/free carnitine. Values are least square means (*n* = 9) in nmol/mg of fresh tissue. The final column is the interaction between clofibrate and age. ^abc^ Means lacking a common superscript differ, (*p* < 0.05). ^+^ Denotes significant linear response.

**Table 5:** Coefficient of correlations between tissues.

	Free Carnitine				Acyl-Carnitine				Total Carnitine			
Correlation	Average	SD	Coefficient	*p*-Value	Average	SD	Coefficient	*p*-Value	Average	SD	Coefficient	*p*-Value
Milk	0.865	0.367			0.511	0.234			1.376	0.585		
-Plasma	0.066	0.029	0.489	0.0001	0.009	0.008	0.374	0.0004	0.075	0.034	0.495	0.0001
-Intestine	0.091	0.052	0.199	0.0627	0.154	0.082	0.215	0.0445	0.244	0.129	0.225	0.0352
-Liver	0.235	0.155	0.167	0.1207	0.245	0.103	0.203	0.0581	0.480	0.244	0.195	0.0691
-Plasma+Liver +Intes tinal Mucosa	0.391	0.176	0.293	0.0062	0.408	0.138	0.299	0.0052	0.800	0.302	0.313	0.0034
Plasma	0.066	0.029			0.009	0.008			0.075	0.034		
-Intestine	0.091	0.052	0.143	0.1853	0.154	0.082	−0.244	0.0221	0.244	0.129	0.064	0.5536
-Liver	0.235	0.155	0.033	0.7626	0.245	0.103	0.011	0.9160	0.480	0.244	0.100	0.3516
-Plasma+Liver +Intestinal Mucosa	0.391	0.176	0.237	0.0264	0.408	0.138	−0.083	0.4447	0.800	0.302	0.221	0.0388
Intestine	0.091	0.052			0.154	0.082			0.244	0.129		
-Liver	0.235	0.155	0.135	0.2034	0.245	0.103	0.098	0.3594	0.480	0.244	0.152	0.1530
-Plasma+Liver +Intestinal Mucosa	0.391	0.176	0.443	0.0001	0.408	0.138	0.662	0.0001	0.800	0.302	0.567	0.0001
Liver	0.235	0.155			0.245	0.103			0.480	0.244		
-Plasma+Liver +Intestinal Mucosa	0.391	0.176	0.935	0.0001	0.408	0.138	0.812	0.0001	0.800	0.302	0.895	0.0001

**Table 6:** Expression of genes *ALD* and *BBH*.

	PPARa Activator *						Age (Day)					Clof *Age
	Clof 0	Clof 0.25	Clof 0.5	SEM	*p*-Value	1	7	14	19	SEM	*p*-Value	*p*-Value
*Mucosa*		Fold				Fold						
*ALD*	0.59	0.61	0.74	0.10	0.517	0.85	0.66	0.49	0.59	0.12	0.253	0.965
*BBH*	2.92	3.51	3.09	0.44	0.614	2.51	3.30	3.25	3.62	0.51	0.494	0.089
*OCTN1*	1.52	1.60	1.38	0.25	0.801	1.62	1.42	1.28	1.69	0.29	0.731	0.491
*OCTN2 * ^#^	1.32 ^a^	3.16 ^b^	1.94 ^ab^	0.52	0.052	1.57	2.27	2.53	2.19	0.62	0.756	0.653
*TMLHE*	0.99	0.89	0.66	0.18	0.381	0.82	0.91	0.64	1.01	0.22	0.638	0.449
*Liver*		Fold					Fold					
*ALD*	1.60	1.63	1.54	0.13	0.878	1.15 ^a^	1.75 ^ab^	1.45 ^a^	2.01 ^b^	0.15	0.0003 ^+^	0.779
*BBH*	2.14	2.08	2.55	0.36	0.595	1.43 ^a^	2.83 ^b^	2.43 ^ab^	2.33 ^ab^	0.42	0.0655 ^^^	0.939
*TMLHE*	8.93	8.66	12.6	1.36	0.076	2.70 ^a^	13.2 ^b^	13.1 ^b^	11.2 ^b^	1.60	0.0001 ^+^^	0.745

* Clofibrate was supplemented to sows during the last week of gestation and the first week of lactation. Clof: clofibrate and 0, 0.25, and 0.5 represent the diet level of clofibrate (% in the feed). ALD, aldehyde dehydrogenase 9 family member A1; BBH, gamma-butyrobetaine hydroxylase (2-oxoglutarate dioxygenase) 1; TML, trimethyllysine hydroxylase; OCTN1, solute carrier family 22 member 4 (SLC22A4); OCTN2, solute carrier family 22 (organic caution/carnitine transporter), member 5 (SLC22A5). Values are least square means (*n* = 9) ± SEM (standard error mean). The final column is the interaction between clofibrate and age. ^ab^ Means lacking a common superscript differ, (*p* < 0.05). ^+^ Denotes significant linear response and ^^^ denotes quadratic response. ^#^ Control vs. clof (*p* = 0.055).

## Data Availability

The raw data supporting the conclusions of this article will be made available by the authors on request.

## Supplementary Materials

The following supporting information can be downloaded at: https://www.mdpi.com/article/10.3390/cimb47121004/s1.

## Abbreviations

The following abbreviations are used in this manuscript:

Ac/Fc	Acyl-carnitine/free carnitine
FA	Fatty acid
OCTN	Organic cation transporter
PPARα	Peroxisome proliferator-activated receptors alpha
