# Supplementary material for: Evaluation of Carnitine Status in Postnatal Piglets from Sows Fed Clofibrate
Source: Curr Issues Mol Biol. 2025 Nov 29;47(12):1004. doi: 10.3390/cimb47121004 (PMC12732076; doi:10.3390/cimb47121004)
Supplement: Supplementary file 1 [file cimb-47-01004-s001.zip › cimb-3981489-supplementary.pdf]

Table S1. Primers used in this study with expected amplicon size post RT-qPCR. Gene Bank numbers demonstrate the genes of the pig genome referenced.

	Forward primer (5'-3'), Sen	Reverse primer (5'-3'), Anti	Amplicon Size, bp	NCBI (Gene Bank)*
BBOX	AACTGGCGGTTACTTCACGG	CCACATCCCAGTCGGCATAA	91	XM_003122909.5
TMLHE	CGGCACACTGACACTACCTATT	AATCCATCTACCAGCAGTGTCC	104	XM_003135511.3
OCTN1	CCCACCCTGGTCAGGAACAT	GGCAGAACTCTGTTGTAAGCAC	110	NM_001145752.1
OCTN2	ATCAGATGCTCAGGGTCAAAGG	CCAGGAGGAAGGAGTCCATTTT	108	XM_013995112.1
ALD	GACTAGCTGCCGGTGTCTTC	AACTCCACAGGGCTGACATT	112	XM_021089487.1
RPL9	GCAACTGTTCGCACCATCTG	CGACGTTGATGGGGAAGTGA	109	NM_001243481.1

*Gene Bank numbers provided to demonstrate the genes of the pig genome referenced.

Table S2. Coefficient of correlations for acyl-carnitine/free carnitine ratio between tissues

	Ac/Fc (acyl-carnitine/free carnitine)			
Correlation	Average	SD	coefficient	*p*-Value
**Milk**	0.613	0.217		
-Plasma	0.141	0.088	0.489	0.504
-Intestine	1.881	0.773	0.015	0.888
-Liver	1.300	0.669	0.255	0.017
-Total	1.123	0.287	0.303	0.004
**Plasma**				
-Intestine			0.315	0.003
-Liver			0.327	0.002
-Total			0.345	0.001
**Intestine**				
-Liver			0.123	0.250
-Total			0.340	0.001
**Liver**				
-Total			0.829	0.0001
